# Parainfluenza Virus 5 V Protein Blocks Interferon Gamma-Mediated Upregulation of NK Cell Inhibitory Ligands and Improves NK Cell Killing of Neuroblastoma Cells

**DOI:** 10.3390/v16081270

**Published:** 2024-08-09

**Authors:** Elisabeth M. Shiffer, Jeremiah L. Oyer, Alicja J. Copik, Griffith D. Parks

**Affiliations:** Burnett School of Biomedical Sciences, College of Medicine, University of Central Florida, Orlando, FL 32827, USA; elisabeth.shiffer@ucf.edu (E.M.S.); jeremiah.oyer@ucf.edu (J.L.O.); alicja.copik@ucf.edu (A.J.C.)

**Keywords:** natural killer cells, parainfluenza virus type 5, immunotherapeutic, interferon-gamma

## Abstract

Natural killer (NK) cells can be effective immunotherapeutic anti-cancer agents due to their ability to selectively target and kill tumor cells. This activity is modulated by the interaction of NK cell receptors with inhibitory ligands on the surface of target cells. NK cell inhibitory ligands can be upregulated on tumor cell surfaces in response to interferon-gamma (IFN-γ), a cytokine which is produced by activated NK cells. We hypothesized that the resistance of tumor cells to NK cell killing could be overcome by expression of the parainfluenza virus 5 (PIV5) V protein, which has known roles in blocking IFN-γ signaling. This was tested with human PM21-NK cells produced through a previously developed particle-based method which yields superior NK cells for immunotherapeutic applications. Infection of human SK-N-SH neuroblastoma cells with PIV5 blocked IFN-γ-mediated upregulation of three NK cell inhibitory ligands and enhanced in vitro killing of these tumor cells by PM21-NK cells. SK-N-SH cells transduced to constitutively express the V protein alone were resistant to IFN-γ-mediated increases in cell surface expression of NK cell inhibitory ligands. Real-time in vitro cell viability assays demonstrated that V protein expression in SK-N-SH cells was sufficient to increase PM21-NK cell-mediated killing. Toward a potential therapeutic application, transient lentiviral delivery of the V gene also enhanced PM21-NK cell killing in vitro. Our results provide the foundation for novel therapeutic applications of V protein expression in combination with ex vivo NK cell therapy to effectively increase the killing of tumor cells.

## 1. Introduction

NK cell cancer immunotherapy is a promising therapeutic approach in which allogenic NK cells can be adoptively transferred to treat cancer [1,2,3]. NK cells are effector lymphocytes of the innate immune system which lyse virus-infected cells and tumor cells [4]. However, there are some challenges in the use of NK cells for tumor therapy, including the increased expression of inhibitory ligands on tumor cells, which can limit NK cell activation and killing. Here, we describe a novel approach to prevent the upregulation of inhibitory ligands on neuroblastoma cancer cells, leading to enhanced in vitro killing by NK cells.

Advances in methods for in vitro NK cell generation have greatly expanded the therapeutic potential of NK cells for cancer immunotherapy. We have previously developed a novel particle-based ex vivo expansion method which yields >1000 fold expansion of highly cytotoxic NK cells [5,6]. This method involves the generation of PM21 particles derived from the plasma membrane of a K562 cell line engineered to express NK cell-stimulating ligands, 41-BBL, and membrane-bound IL-21 [5,6]. Stimulation of PBMCs with PM21 particles results in the expansion of NK cells (PM21-NK cells) with >90% purity and from ~10 to 100 fold greater cytotoxicity than NK cells generated by other conventional methods (i.e., activation with IL2) [5,6]. Further extending their therapeutic potential, PM21-NK cells maintain effector functions following cryopreservation [7]. PM21-NK cells have been shown to be highly effective in reducing tumor burden in mouse models for ovarian cancer [7,8]. PM21-NK cells are currently being tested in early-stage clinical trials (NCT05712278, NCT05115630, NCT04220684, and NCT05726682).

Once activated by recognition of a tumor cell, NK cells can release cytotoxic granules containing perforin and granzyme, causing lysis of the target cell [9]. The activity of NK cells is controlled by the presence of cell surface activating and inhibitory receptors which interact with their respective ligands present on the surface of target cells. The integration of combined signals from activating and inhibitory ligands on target cells can modulate NK cell activation and effector functions, and it can be a major determinant of the effectiveness of NK cell tumor killing [10]. NK cell inhibition is primarily mediated by HLA class 1 binding receptors, including killer immunoglobulin-like receptors (KIRs) and NKG2A. KIRs interact with MHC-I, and CD94-NKG2A heterodimers recognize the non-classical MHC molecule HLA-E [11]. Additionally, some studies reported that NK cell inhibition can also be triggered through the PD-1/PD-L1 axis [12].

The antibody blockade of NK cell inhibitory receptors such as KIRs, NKG2A, and PD-1 is a clinically relevant approach to enhancing the anti-tumor functions of NK cells [8,13,14,15]. Challenges associated with antibody blockade therapy include off-target effects due to a lack of tumor specificity for the antibodies and overcoming the antibody half-life [16,17,18]. An additional challenge is that activated NK cells can release potent cytokines such as interferon-gamma (IFN-γ). After secretion from the NK cell, IFN-γ can bind to its receptor on the surface of tumor cells to initiate a signaling cascade involving signal transduction and activator of transcription 1 (STAT-1) [19], resulting in IFN-γ-mediated increased expression of a large number of genes. This signaling cascade in target tumor cells can result in increased cell surface levels of select NK cell inhibitory ligands such as MHC-I and HLA-E [20,21]. Thus, new approaches which can limit IFN-γ upregulation of cell surface inhibitory ligands on target cells are needed to improve the potency of NK cell therapies.

Combination therapies which integrate NK cells with oncolytic viruses offer unique mechanisms to improve the targeting and killing of tumor cells. A major benefit of using oncolytic viruses is their ability to selectively target tumor cells while leaving normal cells unaffected [22]. A number of paramyxoviruses have been developed as oncolytic vectors, in part due to their ability to modulate anti-tumor immune responses [23,24,25]. We have previously reported that an oncolytic parainfluenza virus can enhance PM21-NK cell-mediated killing of lung cancer cells in cell culture [26]. Parainfluenza virus 5 (PIV5) is quite efficient in modulating host cell antiviral responses, largely due to the activities of the viral V protein [27]. The PIV5 V protein has been shown to slow the progression of the cell cycle [28], a property which is desirable in an oncolytic virus. Most importantly for this study, the PIV5 V protein directs the proteosome-mediated degradation of STAT-1, resulting in cells with a block in IFN-γ signaling and the prevention of new IFN-stimulated gene expression [29].

Given the above properties of PM21-NK cells and the PIV5 V protein, we tested the hypothesis that expression of the V protein in neuroblastoma tumor cells would block IFN-γ-mediated increases in the cell surface expression of NK cell inhibitory ligands and would increase the effectiveness of PM21-NK cell killing. Using real-time cell viability assays, we show that SK-N-SH neuroblastoma cancer cells infected with PIV5 or stably expressing the V protein have an increased rate and extent of PM21-NK cell killing in vitro. Furthermore, PIV5 infection or stable expression of the V protein in cancer cells was found to block IFN-γ-mediated upregulation of NK cell inhibitory ligands. A major benefit of using V protein expression in cancer cells is that upregulation of many IFN-γ-induced NK cell inhibitory ligands can be blocked at once, which otherwise would require combinations of multiple antibodies. Our results provide proof of principle of a novel mechanism to target cancer cells for enhanced NK cell killing.

## 2. Materials and Methods

### 2.1. Cell Lines, Viruses, and Infections

Cultures of SK-N-SH (ATCC) were grown at 37 °C under a humidified 5% CO_2_ atmosphere in Dulbecco modified Eagle medium (DMEM) supplemented with 10% heat-inactivated fetal bovine serum (HI FBS, Gibco, Thermo Fisher Scientific, Waltham, MA, USA). Transduction of SK-N-SH cells using NucLight Red lentivirus (Sartorius, Göttingen, Germany), followed by 0.5 µg/mL puromycin selection, was used to generate SK-N-SH cells expressing a nuclear red fluorescence protein (SK-N-SH NLR cells).

Parainfluenza virus 5 (PIV5) expressing green fluorescence protein (GFP) was grown in MDBK cells and titrated on CV-1 cells as previously described [30]. The cells were infected at a multiplicity of infection (MOI) of 10 unless otherwise indicated. The cells were infected with virus diluted in DMEM with 10% bovine serum albumin (BSA) for 1 h or mock infected with media alone. Following incubation, the cells were washed with phosphate-buffered saline (PBS) and cultured in DMEM supplemented with 2% HI FBS.

### 2.2. Construction of Lentiviral V Protein and Transduction of Stable Cell Lines

A lentivirus expressing the PIV5 V protein was generated by transfection of 293T cells with plasmids encoding the V protein (Vector Builder) and VSV-G envelope (pMD2.G), along with second-generation lentiviral packaging (psPAX2) plasmids. Lentivirus was collected from the supernatant at 24 and 48 h post-transfection. An SK-N-SH cell line stably expressing the V protein was generated by lentivirus transduction in the presence of 0.5 µg/mL polybrene (Millipore Sigma, Burlington, MA, USA), followed by culturing in the presence of 8 µg/mL blasticidin (Invivogen, San Diego, CA, USA).

### 2.3. PM21-NK Cell Preparation and Cryopreservation

PM21 particles were generated, and NK cells were expanded from peripheral blood mononuclear cells (PBMCs) as previously described [5]. The PBMCs were depleted of T cells (EasySep CD3 positive selection kit; STEMCELL Technologies, Cambridge, MA, USA) and then cultured with 100 U/mL interlueukin-2 (IL2, PeproTech, Cranbury, NJ, USA) and 200 µg/mL PM21 particles in SCGM media (Cell Genix) supplemented with 10% non-HI FBS for 7 days. The NK cell cultures post day 7 were maintained in NK cell media consisting of Roswell Park Memorial Institute medium (RPMI) supplemented with 10% non-HI FBS and 100 U/mL IL2. The NK cells were cryopreserved at a concentration of 1 × 10^7^ cells/mL in a solution of 50% RPMI, 40% FBS, and 10% dimethyl sulfoxide (DMSO) as previously described [7].

### 2.4. Cytotoxicity and Cell Killing Assays

Real-time cell viability assays were performed using an IncuCyte instrument (Sartorious, Göttingen, Germany) as previously described [26]. Briefly, target NLR cells were plated in 96 well plates (Corning, Corning, NY, USA) at 7000 cells/well and incubated overnight. For cytokine treatment assays, the cells were washed with PBS and then treated with various concentrations of IFN-γ (PBL Assay Science, Piscataway, NJ, USA) for 18 h. The cells were washed with PBS prior to the addition of NK cells. The plates were incubated at 37 °C within the IncuCyte system and imaged every 2 h using a 10x objective with red, green, and phase channels. The red object count (ROC) corresponding to tumor cell nuclei was calculated for each well. The values for the ROC at each timepoint were expressed as a percentage of the value at time zero (ROC^t0^). The percent cytotoxicity was calculated by normalizing the ROC of the wells containing NK cells plus target cells to the ROC of the wells containing target cells alone in the absence of NK cells.

For transduction experiments, NLR cells were transduced with lentivirus encoding either the blue fluorescence protein (BFP) or V protein as described above. At 2 days post-transduction, the NLR cells were incubated with NK cells, and viability was monitored using the IncuCyte instrument as described above.

### 2.5. Flow Cytometry

Cancer cells were mock infected or infected with PIV5 at an MOI of 10 and incubated at 37 °C overnight. For the cytokine assays, cells were treated for 18 h with varying concentrations of IFN-γ (PBL Assay Science, Piscataway, NJ, USA). Trypsinized cells were stained with conjugated antibodies specific to NK cell inhibitory ligands: PD-L1, MHC-I, and HLA-E (catalog numbers 329708, 311418, and 342606, respectively; BioLegend, San Diego, CA, USA). The cells were quantified by flow cytometry using a CytoFLEX (Beckman Coulter, Brea, CA, USA). CytExpert software (Beckman Coulter, version 2.4) was used to analyze 10,000 independent events.

### 2.6. Western Blotting

The cancer cells were treated as described in the figure legends and were lysed using protein lysis buffer. Cell lysates were resolved on 12% sodium dodecyl sulfate-polyacrylamide gel electrophoresis (SDS-PAGE) gels and transferred to nitrocellulose membranes (Bio-Rad, Hercules, CA, USA). After normalizing the samples with western blotting for β-Actin (1:20,000 dilution, catalog number A5316; Sigma-Aldrich, St. Louis, MO, USA), the samples were then probed with antibodies for the PIV5 V protein (V5 antibody, 1:3500 dilution, catalog number 377,500; Thermo Fisher Scientific, Waltham, MA, USA) and STAT-1 (1:1000 dilution, catalog number 9172; Cell Signaling Technology, Danvers, MA, USA). The blots were visualized using anti-mouse horseradish peroxidase (HRP) conjugated antibodies (Sigma-Aldrich) and chemiluminescence (Thermo Fisher Scientific).

### 2.7. Statistics

Statistical analysis was performed using a GraphPad Student’s *t*-test and two-way ANOVA. In all figures, * indicates a *p* value < 0.05, ** indicates a *p* value < 0.01, *** indicates a *p* value < 0.001, and **** indicates a *p* value < 0.0001.

## 3. Results

### 3.1. PIV5 Established a Productive Infection of Pediatric SK-N-SH Neuroblastoma Cells with Minimal Cytopathic Effects

**Figure 1:**
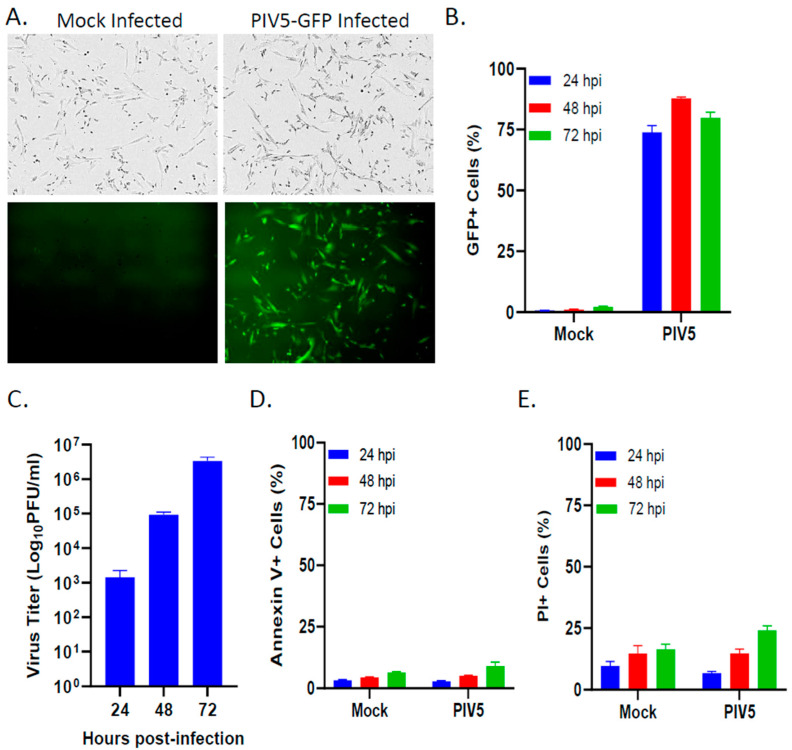
PIV5 established productive infection of pediatric SK-N-SH neuroblastoma cells with minimal cytopathic effects. (**A**,**B**) SK-N-SH cells were mock infected or infected with PIV5 expressing GFP at an MOI of 10 PFU/cell. Cells were examined for GFP expression at 24 hpi with brightfield (BF) or fluorescence (FL) microscopy (**A**) or flow cytometry at 24, 48, and 72 hpi (**B**). (**C**) SK-N-SH cells infected with PIV5 at an MOI of 0.05, with media collected at 24, 48, and 72 hpi, were analyzed for viral titer via a plaque assay. (**D**,**E**) SK-N-SH cells were mock or PIV5 infected at an MOI of 10. At 24, 48, and 72 hpi, cells were analyzed with flow cytometry for annexin V (**D**) and propidium iodide staining (**E**). Values are the means of three replicates, with error bars representing the standard deviation.

To determine the infectivity rates and cytopathic effect of PIV5 in pediatric neuroblastoma cells, the SK-N-SH cells were either mock infected or infected with PIV5 expressing GFP at an MOI of 10. At 24 hpi, the vast majority of the infected cells imaged under brightfield (BF) and fluorescence (FL) expressed GFP, indicating efficient PIV5 infection rates (Figure 1A). Quantification of GFP expression in the PIV5-infected SK-N-SH cells by flow cytometry at 24, 48, and 72 hpi showed that roughly 90% of the cells in the population were infected within 48 hpi (Figure 1B). The infection of SK-N-SH cells with PIV5 was productive, with levels of the infectious progeny virus increasing to >10^6^ fold over 3 days (Figure 1C). To measure the cytopathic effect of PIV5 infection, cell viability was measured through annexin V (Figure 1D) or propidium iodide (PI) (Figure 1E) staining of SK-N-SH cells at 24, 48, and 72 hpi. PIV5 infection resulted in less than 10% of the annexin V^+^ cells (Figure 1D) and less than 25% of the PI^+^ cells within the population (Figure 1E). Taken together, these data indicate that PIV5 efficiently infected the SK-N-SH neuroblastoma cells with minimal cytopathic effects. Thus, due to PIV5 being non-cytopathic in SK-N-SH cells, this model allows for testing of PIV5’s effects on NK cell killing of tumor cells in the absence of direct virus-mediated killing.

### 3.2. PIV5 Infection Enhanced PM21-NK Cell-Mediated Killing of Pediatric Neuroblastoma Cells

**Figure 2:**
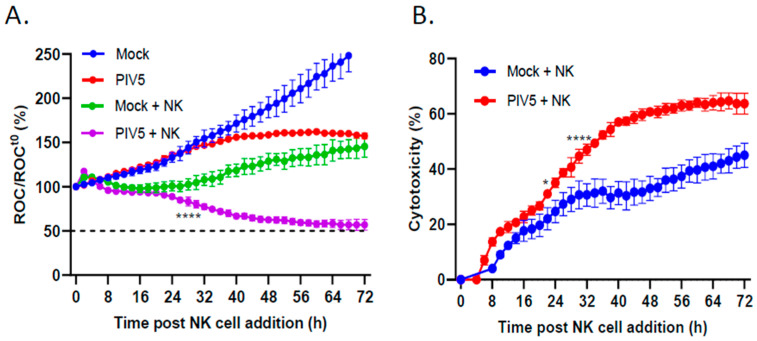
PIV5 infection of SK-N-SH cells enhanced PM21-NK cell-mediated killing. SK-N-SH cells expressing a nuclear red fluorescent protein (SK-N-SH NLR cells) were mock infected or infected with PIV5 at an MOI of 10. At 18 hpi, the mock infected or PIV5-infected cells were incubated with PM21-NK cells at an E:T ratio of 0.625:1. (**A**) Red object count (ROC) per well was recorded on the IncuCyte instrument and normalized to the ROC at time 0 when PM21-NK cells were added and expressed as a percentage. (**B**) Percent cytotoxicity was calculated from the data in panel (**A**). The ROC of the wells containing NK cells plus SK-N-SH NLR cells coincubating together (green and purple curves in panel (**A**), respectively) was normalized to the ROC of the mock infected or PIV5-infected SK-N-SH NLR cells incubated alone without PM21-NK cells (blue and red curves in panel (**A**), respectively). For all panels, values are the means of three replicates, with error bars representing the standard deviation. * and **** indicate when a *p* value < 0.05 or < 0.0001, respectively, first appears on the time course comparing mock infected versus PIV5-infected cells incubated with NK cells, and this statistical significance was maintained through later timepoints.

An IncuCyte instrument was utilized for real-time analysis of how PIV5 infection of the target SK-N-SH cells affected the kinetics of PM21-NK cell-mediated killing. Using SK-N-SH cells which stably expressed a nuclear red fluorescent protein (NucLight Red (NLR)), the IncuCyte instrument could record red fluorescent nuclei as the red object count (ROC) at set intervals and in real time. SK-N-SH NLR cells were mock infected or infected with PIV5 at an MOI of 10, and at 18 hpi, PM21-NK cells were added at an effector-to-target (E:T) ratio of 0.625:1. The ROC per well was determined every 2 h, normalized to the ROC at time zero (ROC^t0^), and expressed as a percentage of the initial time zero when NK cell addition occurred (ROC/ROC^t0^(%)). As shown in Figure 2A, the mock infected SK-N-SH NLR cells continued to proliferate (blue line) with ROC values reaching roughly 250% of time zero. The PIV5-infected cells showed slightly slower growth (red line), consistent with reports that PIV5 infection slows progression through the cell cycle [28]. Incubation of PM21-NK cells with mock infected SK-N-SH NLR cells resulted in plateauing of the ROC over time (green line), which failed to reach 50% of time zero. Most importantly, PIV5 infection resulted in enhanced NK cell killing, which reached 50% of time zero at approximately 72 h after NK cell addition (purple line). Figure 2B shows the same data plotted as the percent of cytotoxicity, where NK cell killing of mock infected and PIV5-infected cells was normalized to values without NK cells to account for the difference in cell growth between the mock and PIV5 infected cells. These data highlight the enhanced killing of PIV5-infected SK-N-SH cells by PM21-NK cells (red line) compared with the killing of mock infected cells (blue line).

### 3.3. PIV5 Infection Inhibited IFN-γ Mediated Upregulation of NK Cell Inhibitory Ligands on the Surface of SK-N-SH Tumor Cells

**Figure 3:**
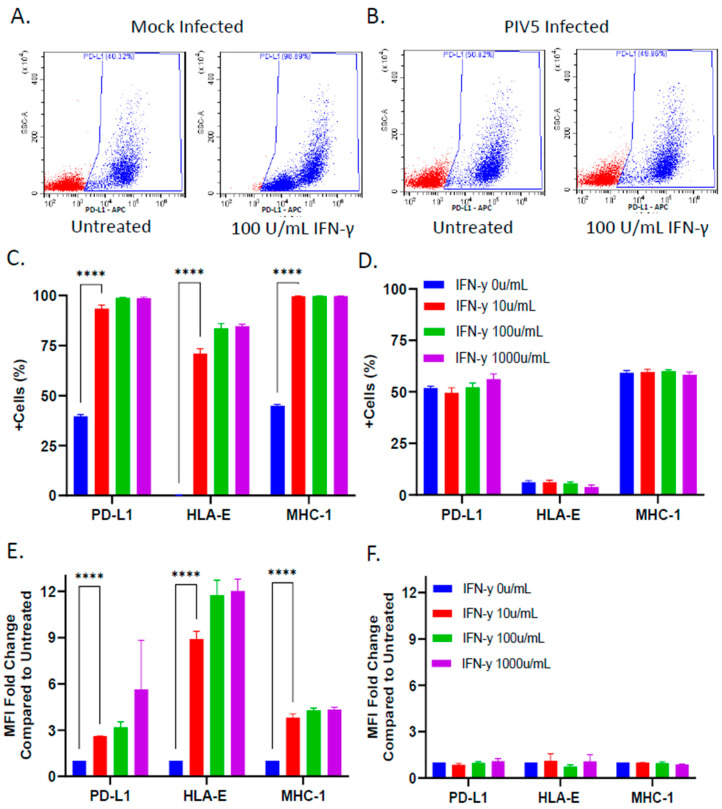
PIV5 infection of SK-N-SH cells blocked IFN-γ-mediated upregulation of cell surface NK cell inhibitory ligands PD-L1, HLA-E, and MHC-I. SK-N-SH cells were mock infected or infected with PIV5 at an MOI of 10. At 18 hpi, the cells were treated with increasing doses of IFN-γ (0, 10, 100, or 1000 units/mL) and incubated for 18 h. The cell surface levels of PD-L1, HLA-E, and MHC-I were determined through antibody staining and flow cytometry. Representative scatterplots of PD-L1 staining of mock infected or PIV5-infected cells are shown in panels (**A**) and (**B**), respectively. The percentage of cells in the populations stained positive for each surface marker and fold change in the mean fluorescence intensity is shown for the mock infected (**C**,**E**) and PIV5-infected cells (**D**,**F**), respectively. Values in all panels are the means of three replicates, with error bars representing the standard deviation. **** indicates a *p* value < 0.0001.

Given that PIV5 infection blocks STAT1-dependent signaling by IFN-γ [27], and IFN-γ has been reported to upregulate some NK cell inhibitory ligands [20,21], we examined the effect of PIV5 infection on the cell surface expression of three prototypic NK cell inhibitory ligands—PD-L1, HLA-E, and MHC-I—following IFN-γ treatment. The mock infected or PIV5-infected SK-N-SH cells were mock treated or treated with IFN-γ for 18 h and analyzed via flow cytometry to quantify the surface expression of PD-L1, HLA-E, and MHC-I. Figure 3A shows typical results from the analysis of PD-L1, where the mock infected cells showed a clear increase in the number of cells with surface expression following IFN-γ treatment. By contrast, the surface expression of PD-L1 did not increase in the case of IFN-γ treatment of the PIV5-infected cells (Figure 3B). For all three ligands, the IFN-γ-treated mock infected cells increased the percentage of positive cells in the population from ~0 to ~70% for HLA-E and from ~40 to ~99% for both MHC-I and PD-L1 (Figure 3C). The mean fluorescence intensity (MFI) fold change compared with untreated samples increased significantly for the SK-N-SH cells stained with fluorescent antibodies against all three NK cell inhibitory ligands (Figure 3E). HLA-E expression increased a maximum of 12 fold in response to IFN-γ treatment, while MHC-I and PD-L1 showed 4 and 6 fold increases, respectively. Most importantly, the IFN-γ-treated, PIV5-infected SK-N-SH cells did not show increases in expression of these surface NK cell inhibitory ligands, with no change in the percentage of positive cells (Figure 3D) or MFI (Figure 3F). Together, these data demonstrate that PIV5 blocked the IFN-γ-mediated upregulation of NK cell inhibitory ligands on the surface of SK-N-SH cells.

**Figure 4:**
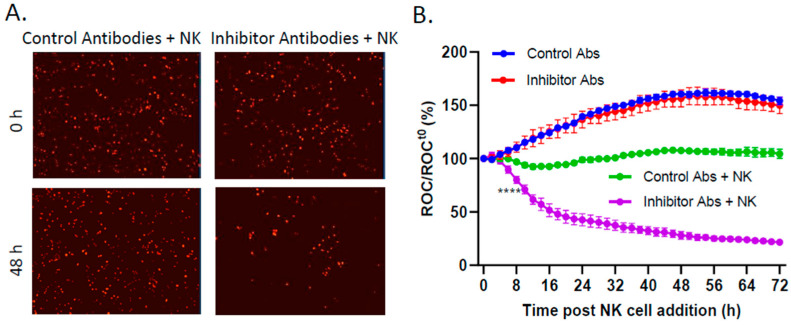
Antibody blocking of NK cell inhibitors PD-L1, HLA-E, and MHC-I enhanced PM21-NK cell-mediated killing. SK-N-SH cells expressing a nuclear red fluorescence protein (SK-N-SH NLR cells) were treated with 100 U/mL IFN-γ for 18 h. Cells were treated with a cocktail of blocking antibodies for PD-L1, HLA-E, and MHC-I or isotype control antibodies for 1 h before the addition of PM21-NK cells at an E:T ratio of 0.625:1 in the presence of antibodies. (**A**) Representative red fluorescent images of SK-N-SH NLR cells incubated with isotype control or inhibitory antibody were recorded using the IncuCyte instrument at 0 and 48 h post NK cell addition. (**B**) Red object count (ROC) per well was quantified using the IncuCyte instrument and normalized to the ROC at time 0 (ROC/ROC^t0^) when NK cells were added, expressed as a percentage of time 0. Values are the means of three replicates, with error bars representing standard deviation. **** indicates when a *p* value < 0.0001 first appeared on the time course, comparing inhibitor antibody-treated verses isotype control-treated cells incubated with NK cells, and this statistical significance was maintained at later timepoints.

We used antibody blocking to test the functional contribution of surface expression of these three prototypic NK cell inhibitory ligands—PD-L1, HLA-E, and MHC-I—on the killing of SK-N-SH tumor cells with PM21-NK cells. The target SK-N-SH NLR cells were mock treated or treated with IFN-γ for 18 h, followed by incubation with a cocktail of antibodies specific to PD-L1, HLA-E, and MHC-I. After the addition of PM21-NK cells, changes in the ROC were monitored by IncuCyte every 2 h. As shown in the micrographs in Figure 4A, the samples containing anti-inhibitor antibodies showed a clear decrease in the ROC by 48 h post PM21-NK cell addition. As shown in the time course in Figure 4B, the SK-N-SH NLR target cells which received isotype control antibodies (blue line) proliferated to the same extent in the absence of PM21-NK cells as the samples which received the inhibitory ligand antibodies (red line). Most importantly, the SK-N-SH NLR target cells treated with inhibitory ligand antibodies showed a rapid ROC loss in the presence of PM21-NK cells (purple line), reaching 50% of time zero by 16 h, compared with the cells which received control antibodies (green line). These data indicate that these three NK cell inhibitory ligands—PD-L1, HLA-E, and MHC-I—together can limit PM21-NK cell killing of SK-N-SH cells.

### 3.4. PIV5 V Protein Alone Was Sufficient to Block IFN-γ-Mediated Upregulation of NK Cell Inhibitory Ligands

**Figure 5:**
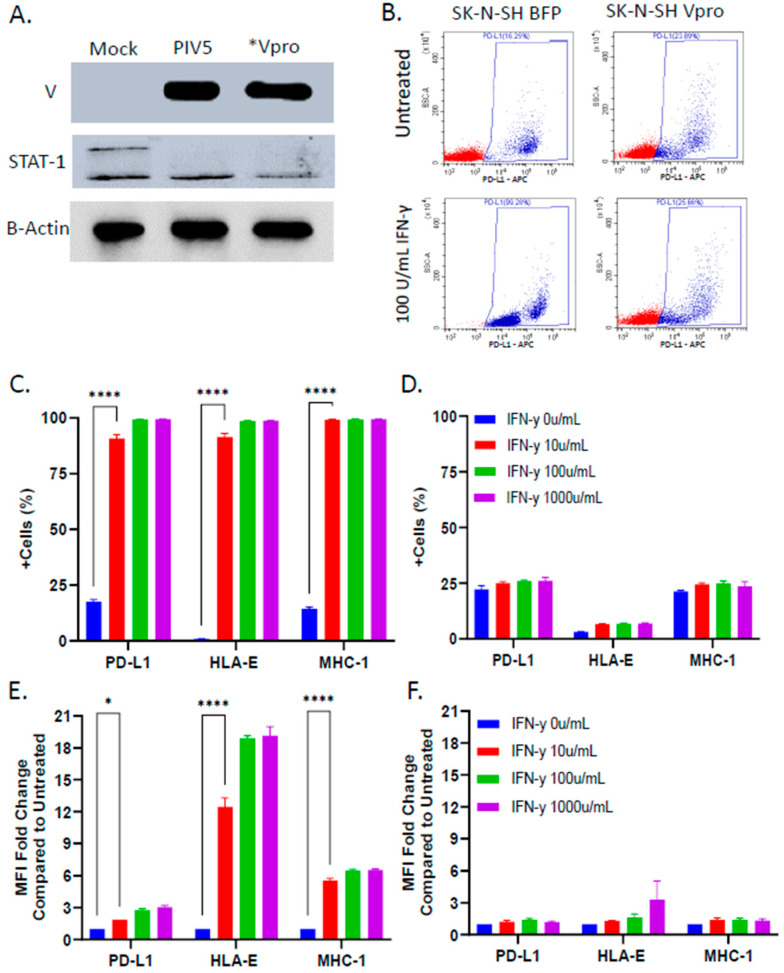
Stable expression of the PIV5 V protein in SK-N-SH neuroblastoma cells conferred resistance to IFN-γ-mediated upregulation of NK cell inhibitory ligands. (**A**) Lysates from mock infected, PIV5-infected, or V protein-expressing SK-N-SH cells were analyzed through western blotting for levels of V protein and STAT-1. * For V protein staining, lysates of V protein expressing cell lines were loaded at 3 times the normalized amount. (**B**–**F**) SK-N-SH cells expressing BFP or the V protein were treated with increasing doses of IFN-γ (0, 10, 100, or 100 U/mL) for 18 h. Cell surface levels of PD-L1, HLA-E, and MHC-I were determined with antibody staining and flow cytometry. Representative scatterplots of PD-L1 staining of naïve or V protein-expressing cells treated with 100 units/mL IFN-γ are shown in panel (**B**). The percentage of cells in the population staining positive for each surface marker and fold change in mean fluorescence intensity are shown for BFP (**C**,**E**) and V protein-expressing cells (**D**,**F**), respectively. Values in all panels are the means of three replicates, with error bars representing the standard deviation. * and **** indicate a *p* value < 0.05 and < 0.0001, respectively.

Given its role in blocking IFN signaling [27], we tested the hypothesis that expression of the V protein alone was sufficient to prevent IFN-γ-mediated upregulation of NK cell inhibitory ligands. An SK-N-SH cell line was generated to stably express the PIV5 V protein (Vpro). Cells expressing blue fluorescence protein (BFP) were also generated as a control for lentiviral transduction. As shown in Figure 5A, western blotting for the V protein and STAT-1 showed that the Vpro cells expressed V protein and had reduced levels of STAT-1, similar to that seen with bone fide PIV5-infected SK-N-SH cells.

To determine the role of the V protein in mediating NK cell ligand expression on target cells, the BFP-expressing control cells or V protein-expressing cells were treated with IFN-γ and then analyzed for surface expression of NK cell inhibitory ligands PD-L1, HLA-E, and MHC-I. As shown in the representative data for PD-L1 in Figure 5B, IFN-γ treatment of BFP control cells increased the percentage of cells in the population that were positive for PD-L1, whereas there was no change in surface expression in the case of the IFN-γ-treated Vpro cells. For all three ligands, analysis of the IFN-γ-treated BFP-expressing cells showed an increase in the percentage of positive cells in the population from ~0 to ~90% for HLA-E and from ~10 to ~95% for both MHC-I and PD-L1 (Figure 5C). The MFI fold change compared with the untreated samples increased significantly for all three ligands (Figure 5E). In stark contrast, the Vpro cells showed no increase in the percentage of positive cells expressing these NK cell inhibitors and little to no increase in the MFI following IFN-γ treatment (Figure 5D,F). These data demonstrate that expression of the PIV5 V protein alone was sufficient to block IFN-γ-induced upregulation of select NK cell inhibitory ligands on the surface of SK-N-SH cells.

### 3.5. PIV5 V Protein Alone Was Sufficient to Enhance NK Cell-Mediated Killing and Overcome IFN-γ-Induced Reduction in NK Killing

**Figure 6:**
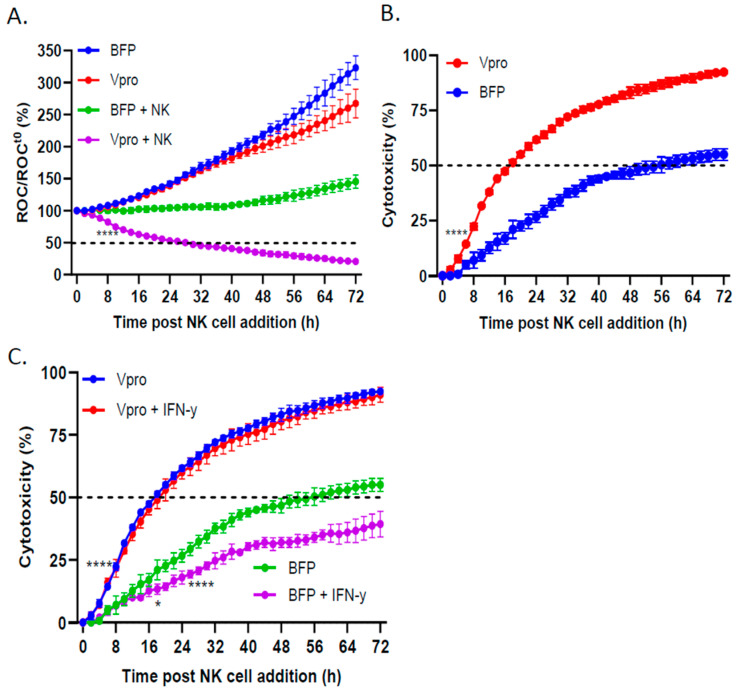
PIV5 V protein expression in SK-N-SH cells increased PM21-NK cell-mediated killing. (**A**,**B**) SK-N-SH NLR cells stably expressing BFP or V protein were incubated with PM21-NK cells at an E:T ratio of 0.625:1. (**A**) The ROC per well was recorded on the IncuCyte instrument and normalized to the ROC at time 0 when PM21-NK cells were added, expressed as a percentage (ROC/ROC^t0^(%)). (**B**) Percent cytotoxicity was calculated from the data in panel (**A**). The ROC of wells containing NK cells plus BFP- and V protein-expressing SK-N-SH NLR cells coincubated together (green and purple curves in panel (**A**), respectively) was normalized to the ROC of BFP- and V protein-expressing SK-N-SH NLR cells incubated in the absence of PM21-NK cells (blue and red curves in panel (**A**), respectively). (**C**) BFP- or V protein-expressing cells were left untreated or treated with 100 U/mL IFN-γ for 18 h. PM21-NK cells were added at an E:T ratio of 0.625:1. Percent cytotoxicity was calculated by normalizing the ROC/ROC^T0^ of BFP- or V protein-expressing cells when incubated with PM-21 NK cells compared with ROC/ROC^T0^ in the absence of NK cells. Values are the means of three replicates, with error bars representing standard deviation. * and **** indicate when a *p* value < 0.05 and < 0.0001, first appeared on the time course, respectively, comparing BFP versus Vpro cells incubated with NK cells, and this statistical significance was maintained at later timepoints.

To determine the effect of V protein expression in the target cells on mediating NK-cell killing, BFP control or Vpro cells expressing NLR were incubated with NK cells, and cell viability was monitored using the IncuCyte instrument. As shown in Figure 6A, the BFP (blue line) and Vpro (red line) cells both continued to proliferate, reaching roughly 300% of time zero within 72 h. NK cell killing of the BFP control cells (green line) was relatively low, with the percentage of ROC/ROC^t0^ never reaching less than 100%. In sharp contrast, NK cell killing of Vpro cells (purple line) reached 50% of time zero by 24 h after NK cell addition. When expressed as the percent of cytotoxicity (Figure 6B), both the rate and extent of NK cell killing were significantly enhanced in the Vpro cells compared with the BFP control cells. Together, these data demonstrate that the expression of V protein alone in target cells was sufficient to enhance NK cell killing.

We tested the hypothesis that IFN-γ treatment of control SK-N-SH cells would decrease NK cell killing (consistent with increased expression of inhibitory ligands) and that V protein expression would prevent this IFN-γ-mediated reduction in killing. BFP and Vpro cells were left untreated or treated with IFN-γ before the addition of PM21-NK cells, and the ROC was monitored over time using an IncuCyte instrument. As shown in Figure 6C, the BFP control cells (green line) were killed by PM21-NK cells, reaching 50% cytotoxicity at ~52 h post NK cell addition. Consistent with the effects of IFN-γ increasing inhibitory ligand expression, the IFN-γ-treated BFP cells (purple line) were less susceptible to PM21-NK cell killing, failing to reach 50% cytotoxicity. In contrast, PM21-NK cell killing of Vpro cells (blue line) was quite efficient, reaching 50% cytotoxicity within 16 h post NK cell addition, and IFN-γ treatment did not alter the kinetics or extent of PM21-NK cell killing (red and blue overlapping lines, respectively). Together, these data suggest that the PIV5 V protein was sufficient to enhance NK cell killing of SK-N-SH tumor cells and was capable of overcoming an IFN-γ-induced decrease in NK cell killing.

### 3.6. Lentiviral Delivery of V Protein Enhanced NK Cell-Mediated Killing of Tumor Cells

**Figure 7:**
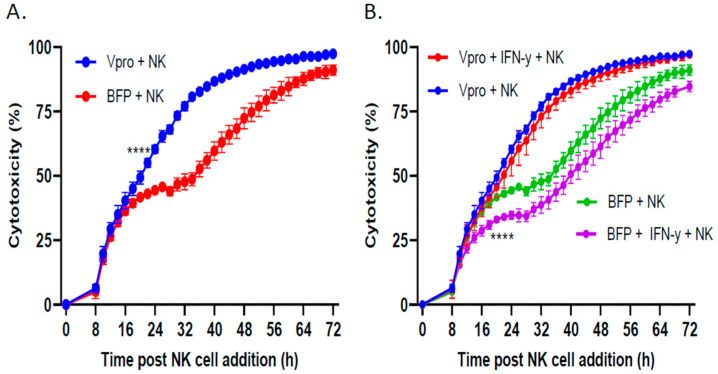
Lentiviral delivery of PIV5 V protein increased tumor cell killing by PM21-NK cells. SK-N-SH NLR cells were transduced with lentivirus expressing either the PIV5 V protein or BFP as a control and incubated for 2 days. (**A**,**B**) Cells were left untreated (**A**) or treated with 100 U/mL IFN-γ (**B**) and incubated for 18 h before the addition of PM21-NK cells at an E:T ratio of 2.5. An IncuCyte instrument was used to monitor red fluorescence in real time. The percent cytotoxicity was calculated by normalizing the ROC/ROC^T0^ of the BFP- or V protein-expressing cells when incubated with PM-21 NK cells compared with the control ROC/ROC^T0^ in the absence of NK cells. Values are the means of three replicates, with error bars representing standard deviation. **** indicates when a *p* value < 0.0001 first appeared on the time course, comparing BFP versus Vpro transduced cells incubated with NK cells, and this statistical significance was maintained at later timepoints.

For therapeutic utility, the V protein would need to be delivered to tumor cells in situ to sensitize them to killing via adoptively transferred NK cells. As a proof of concept, we used lentivirus to transiently transduce SK-N-SH cells to express the V protein or BFP control without selection for stable cell lines. PM21-NK cells were incubated with cells transiently transduced to express the BFP or V protein, and cell viability was measured by an IncuCyte instrument. As shown in Figure 7A, the cells which received lentiviral delivery of the V protein (blue line) were more susceptible to PM21-NK cell killing compared with the cells which received lentiviral delivery of the control BFP protein (red line). Furthermore, IFN-γ treatment of tumor cells which received BFP lentiviral transduction resulted in a slight reduction in PM21-NK cell killing (Figure 7B, purple line) compared with the untreated cells (green line). In contrast, lentiviral delivery of the V protein resulted in no change in PM21-NK cell killing when comparing the IFN-γ-treated (red line) and non-treated cells (blue line). These results provide a proof of concept for the therapeutic potential of V protein expression in combination with adoptive NK cell therapy.

## 4. Discussion

NK cell-based cancer immunotherapies provide a powerful approach for reducing tumor burden. Adoptive NK cell therapy as an anti-cancer therapeutic is currently being evaluated in clinical trials for a variety of malignancies, including neuroblastoma, lung, and breast cancers [31]. However, one potential limitation of this cell-based therapeutic is that IFN-γ released from activated NK cells can induce STAT-1-dependent expression of NK cell inhibitory ligands on the surface of the cancer cells being targeted for NK cell killing [21]. While antibody blocking of inhibitory ligands or STAT inhibitors has been shown to be effective at enhancing NK cell lysis of tumor cells, these modalities have some potential limitations, including off-target effects on non-cancerous cells and the activation of other immune cells. Thus, there is a need for alternative or complementary approaches which target the cancer cell to block induction of inhibitory ligands. Here, we described an alternative approach which has the advantage of blocking upregulation of multiple NK cell inhibitory ligands, whereas combinations of multiple antibodies would be necessary to achieve the same effect using conventional antibody blockade therapy.

NK cell activity is regulated by a collection of inhibitory and activating receptors which interact with their respective ligands on the surface of target cells to modulate NK cell killing [32,33]. The inhibitory ligands PD-L1, HLA-E, and MHC-I are of particular importance in mediating NK cell killing of tumor cells by interacting with their respective receptors—PD-1, CD94/NKG2A, and KIRs—on the surface of the NK cells [14,34,35]. Furthermore, IFN-γ-mediated upregulation of these NK cell inhibitory ligands on cancer cells is associated with decreased NK cell killing and poor therapeutic prognosis [8,20,21,36]. Consistent with these findings, we have shown for pediatric SK-N-SH neuroblastoma cells that antibody blocking of cell surface PD-L1, HLA-E, and MHC-I can increase PM21-NK cell killing (Figure 4). Likewise, IFN-γ treatment of SK-N-SH cells increased the surface expression of these inhibitors (Figure 3).

The use of oncolytic viruses is a promising cancer therapeutic modality, based on the ability to directly target tumor cells as well as activate the adaptive and innate immune systems [37,38,39]. Furthermore, oncolytic viruses have been shown to enhance NK cell-mediated killing of cancer cells [26,40]. Given that PIV5 infection results in a blockage of IFN signaling [30,41,42], we tested the hypothesis that PIV5 infection of SK-N-SH cells could block IFN-γ-mediated upregulation of NK cell inhibitory ligands on the surface of tumor cells, thereby enhancing NK cell-mediated killing. We showed that PIV5-infected cells did not respond to IFN-γ treatment, resulting in unchanged cell surface expression of the inhibitory ligands PD-L1, HLA-E, and MHC-I (Figure 3). This observation provides a potential explanation for the increase in PM21-NK cell killing following PIV5 infection of SK-N-SH neuroblastoma cells (Figure 2). While PIV5 is largely noncytopathic toward the tumor cell itself [43,44,45], we have shown previously that PIV5 infection contributes additional signals for PM21-NK cell killing of lung cancer cells through cell surface expression of the viral hemagglutinin-neuraminidase (HN) glycoprotein [26], which is recognized by NK cell receptor NKp46 [46]. Thus, an oncolytic PIV5 vector can both contribute positive signals (HN) to NK cell activity and block the upregulation of negative signals via the V protein. Future work will focus on the interaction between NK cells and tumor cells to understand the molecular engagements which are important in enhancing NK cell killing and how PIV5 infection or V protein expression might alter these interactions.

Using a stable cell line expressing the PIV5 V protein, we demonstrated that V protein expression alone, in the absence of the rest of the PIV5 infection, is sufficient to block IFN-γ-induced upregulation of the NK cell inhibitory ligands PD-L1, HLA-E, and MHC-I (Figure 5) and was also sufficient to enhance PM21-NK cell-mediated killing (Figure 6). For therapeutic applications, the V protein would need to be delivered by a vector or other modalities directly to the tumor site. Here, using transient lentivirus delivery as our proof of concept, we showed that V protein expression was sufficiently high to improve PM21-NK cell-mediated killing of SK-N-SH neuroblastoma cells when delivered transiently without selection for stable cell lines (Figure 7). While V protein delivery is successful in enhancing NK cell killing of primed tumor cells in vitro, validation in animal models is necessary to determine the efficacy of this approach under more complex variables. Since evidence suggests that the V protein acts catalytically to dismantle STAT-1 signaling [47], it is likely that the delivery of only small amounts of V protein will have dramatic effects on increasing NK cell lysis of cancer cells. Other cancer gene therapy delivery systems (e.g., adeno-associated virus) should be explored to potentially expand the range of tumor cells which could be treated using V protein delivery and NK cell combination therapy. For example, non-viral vectors such as lipid-based nanoparticles have been shown to effectively deliver genes across the blood–brain barrier to reach glioma tumors [48,49].

V protein expression can prevent IFN-γ-induced emergence of cancer cells which are resistance to NK cell killing. It is important to note that how effective V protein can be in enhancing NK cell killing will be determined by both the basal and IFN-γ-induced levels of NK cell inhibitory ligands on the surface of a particular cancer cell type. For example, in the case of cancer cells with high basal levels of NK cell inhibitory ligands, the effect of V protein delivery on increasing NK cell killing would be expected to be low compared with the effect of V protein expression on cancer cells with low levels of inhibitor expression. Likewise, the degree to which a given cancer cell responds to IFN-γ to increase NK cell inhibitor expression will determine the effectiveness of the V protein in enhancing NK cell killing. Thus, since the V protein blocks upregulation of inhibitors by blocking IFN-γ signaling, a rational approach for the therapeutic benefit of V protein would be to prescreen cancer cells to determine the level and type of NK cell ligands present. Future work will focus on determining the relationship between the basal level and landscape of NK cell inhibitory ligands on a range of cancer cell lines versus the effectiveness of V protein enhancement of NK cell killing.

In addition to IFN-γ, activated NK cells also release tumor necrosis factor-α (TNF-α) [9], which has been shown to upregulate the expression of inhibitory ligands PD-L1 and MHC-I in various tumor cell types [50,51,52]. This raises the question of whether blocking IFN signaling alone is the most effective mechanism to regulate NK cell inhibitory ligand expression on the surface of tumor cells. Targeting TNF-α signaling alone or in combination with IFN-γ signaling could provide greater modulation of ligands and thereby more effective NK cell killing. It is known that different paramyxoviruses express V proteins which modulate cellular regulatory pathways through different mechanisms. For example, PIV2 blocks toll-like receptor 7 (TLR7) and TLR9 signaling, which are critical components of the TNF-α signaling pathway [53]. Other paramyxoviruses use alternative mechanisms of preventing IFN signaling which may be more effective than the PIV5 V protein, such as Nipah virus, which forms high molecular weight complexes with STAT-1 and STAT-2 [54]. These alternative mechanisms might prove to be effective alternative approaches to altering tumor cells to make them more sensitive to NK cell killing. There is great therapeutic potential to engineer other V protein vectors and determine how these can be used to further enhance NK cell immunotherapy.

## Data Availability

The raw data supporting the conclusions of this article will be made available by the authors on request.
